# Bathing in Atopic Dermatitis in Pediatric Age: Why, How and When

**DOI:** 10.3390/pediatric16010006

**Published:** 2024-01-08

**Authors:** Margherita Pagliaro, Luca Pecoraro, Camilla Stefani, Sara Pieropan, Giorgio Piacentini, Angelo Pietrobelli

**Affiliations:** 1 Pediatric Unit, Department of Surgical Sciences, Dentistry, Gynecology and Pediatrics, University of Verona, 37126 Verona, Italycamilla.stefani@studenti.univr.it (C.S.); angelo.pietrobelli@univr.it (A.P.); 2 Pennington Biomedical Research Center, Baton Rouge, LA 70808, USA

**Keywords:** atopic dermatitis, bathing, bathing additives, bleach baths, baby cleansers, bath oils, salt bath, natural additives

## Abstract

Atopic dermatitis is a chronic inflammatory skin disease. The treatment plays an important role in influencing the patients’ quality of life. The basic management consists of appropriate skin cleansing, including bathing and eventually using bathing additives. Recommendations regarding frequency and duration of bathing, water temperature and usefulness of bathing additives are widely different, often leading to confusion among patients. This review aims to give insights into the best bathing practices and the use of bathing additives in atopic dermatitis in children. Several bathing additives, including bleach baths, commercial baby cleansers, bath baby oils and bath salt, appear to be promising adjunctive therapies for atopic dermatitis due to their anti-inflammatory, anti-bacterial, anti-pruritus and skin barrier repair properties through different mechanisms of action. However, their efficacy and safety are not fully understood in some cases. The usefulness of other bath additives, such as acidic and more natural substances (green tea extracts, pine tar, sodium bicarbonate), is still under investigation. Further studies are needed to determine their optimal use to achieve clinical benefit safely.

## 1. Introduction

Atopic dermatitis (AD) is a chronic inflammatory skin disease that can significantly impact the quality of life of patients and their families. It is the most common skin disease in children, affecting up to 20% worldwide [1]. Given its chronic course, treatment is an important aspect of the disease that will accompany patients throughout their lives. A complex and multifactorial etiopathogenesis characterizes AD. Genetic susceptibility plays a major role, leading to different alterations in the skin barrier, microbial dysbiosis [2] and immunologic dysregulation [3]. Alteration in the skin barrier, including abnormal lipid metabolism and altered epidermal structural protein, such as filaggrin and protease inhibitors, can lead to increased transepidermal water loss [TEWL], increased pH [3] and consequent susceptibility to infections and sensitization to aeroallergens [4]. The innate immune system of the skin is impaired in patients with AD, characterized by an imbalance of T-helper type 2 [Th2] to Th1 cytokines, resulting in a high IgE-mediated hypersensitivity. The interaction between pathogens and the skin’s immune cells plays an important role in inflammation, activating an inflammatory cycle and a higher susceptibility to bacterial infection [3]. Environmental triggers such as allergens, skin irritants, infections and physical irritants (e.g., tobacco smoke, traffic exhaust, extreme temperatures, cold or humidity) can also exacerbate AD symptoms [5,6,7]. The diagnosis of AD is made based on clinical presentation and family history. No gold standard laboratory test for AD exists [8]. The distribution of lesions differs depending on age, with infants and young children typically affected on the face and extensor surfaces of extremities. These features are followed by allergic rhinoconjunctivitis, food allergies and asthma [“atopic march”] [9]. The main feature of the atopic skin is the severe xerosis. The lesions display a heterogeneous course that includes both acute and chronic phases. The former is characterized by itchy, relapsing, eczematous lesions, erythema, vesiculation, oozing and exudation, characterized by lichenification and desquamation. Infections like bacterial, viral and fungal infections are common AD complications. The atopic skin is massively colonized with Staphylococcus aureus [*S. aureus*] in damaged and non-damaged skin, aggravating the inflammatory skin condition [10,11]. After the diagnosis, the first step is correctly assessing disease severity, which is crucial for selecting and monitoring treatment response [8,12,13,14]. Different instruments are used to establish AD severity. The classical composite score is the ‘Scoring of Atopic Dermatitis’ (SCORAD), which evaluates AD signs and symptoms. The second most commonly used tool is the Eczema Area and Severity Index (EASI), which only considers clinical signs, excluding subjective symptoms. Finally, the Patient-Oriented Eczema Measures for Eczema [POEM] is a symptoms-only score to measure subjective symptoms but not objective signs [8]. Regarding AD monitoring, it has been demonstrated that elevated TEWL in early infancy may identify patients at high risk of developing AD [15,16]. TEWL measurement can be considered sufficiently accurate for disease monitoring [17]. The main goal of AD treatment is to relieve the symptoms, improve skin barrier function and prevent complications. It is important for patients and their caregivers to have a clear picture of the disease and to understand which existing therapeutic strategy is more suitable for their symptoms. Recently, numerous guidelines have been published for atopic dermatitis management in children [8,12,13,14]. The treatment regimen for AD is complex, encompassing a variety of approaches to effectively manage the condition. One of the main steps consists of appropriate skin cleansing followed by applying emollients. Emollients help hydrate the skin’s SC and reduce TEWL [8,12]. The effectiveness of the application of emollients to prevent AD was recently confirmed to be better used once daily than twice daily [18]. Topical corticosteroids [TCS] are essential to treat acute flares. In mild and severe AD cases, systemic treatment with immunosuppressants and targeted immunomodulating agents may represent a successful alternative [19,20,21,22]. In conclusion, the treatment of AD follows a multistep approach that is tailored according to disease severity. There is some evidence of the importance of bathing in the context of the first step of AD management. At the same time, contradictory statements about bathing frequency, duration and timing for other treatments are known. This event can confuse the patient and the caregivers. Some guidelines explore using bathing additives to relieve symptoms, but there is limited research on the use of bathing additives in treating AD. Skin cleansing represents the primary aim of topical treatment of AD, but it must be done properly to avoid the disruption of the skin barrier, worsening symptoms and facilitating bacterial proliferation. Studies show that prolonged exposure to water might lead to disruption of the stratum corneum [SC], leading to increased skin permeability and susceptibility to irritants and infections [23]. In addition, TEWL can occur through evaporative losses after bathing, worsening skin dryness and irritation [24]. In order to obtain the maximum therapeutic effect, in recent years, physicians have tried to identify the optimal bathing parameters such as duration, frequency, temperature of water and the use of water alone or with additives. This paper aims to provide a comprehensive overview of the current evidence on bathing to provide a clearer picture of the basic management of AD in childhood.

## 2. Duration, Frequency and Temperature

Most international guidelines recommend bathing daily for about 5–10 min with warm water and using emollients immediately after bathing. However, there is some variation in the specific recommendations in the context of atopic dermatitis in pediatric age (Table 1). 

The European guidelines [8] recommend only a short duration (5 min) with the use of bath oils in the last 2 min of bathing [temperature of water 27–30 °C]. There are no recommendations about the weekly frequency. An update in the European guideline [13] reports that a small, randomized study regarding the frequency of bathing procedures did not show any difference between twice-weekly versus every day [25]. Eichenfield et al. recommended up to once-daily bathing to remove serous crust and the subsequent administration of moisturizers [12]. Finally, the Korean consensus guidelines [14] specify that bathing should not exceed once per day. Emerging research suggests that the frequency of bathing may not significantly impact the severity of AD. A systematic review by Tammy Hua and colleagues found that bathing or showering more than 7 times per week versus less than 7 times per week was not associated with significant differences in EASI score [25]. Similar results were obtained in a prospective longitudinal cohort study conducted in 2023. The authors concluded that showering or bathing less than daily versus once daily was not associated with different outcomes, but showering/bathing more than daily versus once daily was associated with higher SCORAD, EASI and POEM scores [26]. These findings suggest that the frequency of bathing did not seem to play an important part in managing AD, although maintaining hydration is more important [27]. A recent study by Uros Rakita showed that consistent and even inconsistent application of moisturizer after showering/bathing was associated with lower severity scores [26]. Regarding the water temperature, guidelines mainly recommend bathing with warm water. For instance, the European guidelines [8] recommend a temperature of about 27–30 °C, while the Japanese guidelines [28] recommend a temperature of 36–40 °C. The rationale for this recommendation is that itching is induced at a skin temperature of 42 °C or higher, while 36–40 °C is the optimum range for recovery of skin barrier functions. 

**Table 1:** Comparison between international guidelines about way of bathing in atopic dermatitis in children.

	AAD [12]	JTF [21]	European Guidelines [8]	EuroGuiDerm [13]	Japanese Guideline [28]	Korean Guideline [14]
Bathing time	No standard for duration: short periods (5–10 min)	At least 10 min	Short duration (only 5 min) + use bath oils (in the last 2 min of bathing)	Short duration (only 5 min) + use bath oils (in the last 2 min of bathing)	No clear indication (depends on the patient and the symptoms”)	Short duration (5–10 min)
Weekly/daily frequency	No standard for frequency: it is generally recommended to do up to once daily bathing.	\	\	No difference in bathing daily versus twice weekly	\	Not exceed once per day
Temperature of bath water	Warm water (no mention of degree centigrade)	Warm water (no mention of degree centigrade)	27–30 °C	The water temperature should not be too high (no mention of degree centigrade)	36–40 °C	27–30 °C

## 3. The Use of Additives

While bathing additives gained popularity in patients with inflammatory skin conditions, their clinical benefit is still unclear [12]. Bathing additives are believed to provide therapeutic benefits for AD when used with mainstay treatment modalities [29]. The BATHE study found no evidence of a clinical benefit from emollient bath additives in addition to standard eczema management [30]. At the same time, Maarouf et al. demonstrated that some bathing additives, such as dead sea salt, oatmeal or natural oils, may improve skin condition and reduce the need for pharmacological treatments [29]. Several differences among the guidelines exist. The Japanese Task Force [JTF] [21] and Asia-Pacific [31] groups suggest that adding additives to the normal routine of bathing may have beneficial effects. On the other hand, the American Academy of Dermatology [AAD] group [12] does not recommend their use because of insufficient evidence, except for bleach baths in patients with recurrent skin infections. The European Guidelines [8] do not deepen this topic. Several bathing additives have emerged as promising adjunctive therapies, including bleach baths, commercial baby cleansers, bath baby oils, bath salt and more natural products [29] (Table 2).

### 3.1. Sodium Hypochlorite (Bleach Baths)

Bleach baths [dilute sodium hypochlorite (NaOCl 0.005%)] represent a promising treatment for AD, but there is still some debate about their use. A recent systematic review and meta-analysis of 307 patients with moderate-to-severe AD demonstrated that dilute bleach baths decreased about 22% of the AD severity [32]. The clinical benefit of NaOCl is primarily attributed to its antimicrobial activity [33,34,35]. Recent evidence suggested that the antimicrobial effect is probably obtained at higher concentrations than those used in AD treatment [36]. A study by Rathore et al. [37] found that a 2.5 μL/mL dilution of bleach, equivalent to one-half cup of bleach in a one-quarter-filled bathtub, had a significant effect on killing the community-associated methicillin-resistant *S. aureus* [CA MRSA] in vitro. Similar ex vivo studies found that bleach concentrations greater than 0.03% were required to eradicate *S. aureus* biofilm, although those levels are cytotoxic to human cells and should not be used clinically [38,39]. Multiple studies suggested that NaOCl has other effects besides the antimicrobial, including modulating the surface microbiome without causing antibiotic resistance [40] and reducing the secretion of inflammatory cytokines in affected skin tissue, leading to anti-pruritogenic and anti-inflammatory effects [41]. A recent study conducted by Stolarczyk and colleagues shows that bleach baths in atopic patients enhance skin barrier and reduce itch but do not normalize skin dysbiosis or reduce *S. aureus* [42]. Although bleach baths seem efficacious for clinical improvement in AD, their benefit over water baths alone remains unclear. A review suggests no significant difference in AD frequency for bleach baths versus water baths alone and no differences in *S. aureus* density [43]. Furthermore, there are several considerations about the safety of sodium hypochlorite. Less common but more serious adverse effects reported in a few cases can include asthma exacerbations due to inhalation of fumes from bleach baths [44]. The hospitalization events were associated with noncompliance and the exacerbation of *S. aureus* infection [35,45]. Nevertheless, the most common adverse effects are mild and temporary, such as dry skin and irritation due to its alkaline pH [46]. A recent study about the clinical toxicology of sodium hypochlorite concluded that brief skin exposure to household bleach normally causes only minimal and transient effects and household bleach’s accidental ingestion is rarely of clinical importance [46]. The current AAD [12], Asia-Pacific [31] and European guidelines [8] endorse the use of diluted bleach baths, especially in patients with moderate to severe AD with frequent bacterial infections. In contrast, the JTF guideline [21] states that assessing definitive statements on using bleach baths is impossible.

### 3.2. Baby Cleansers

There is no consensus on using cleansers in the skincare of patients with AD. They are commonly used to facilitate grease and dirt stripping. There are three types of cleansing agents: soaps, synthetic detergents (syndets) and lipid-free cleansing agents. Most soaps have an alkaline pH that can worsen the typical higher pH of atopic skin and enhance the protease function, leading to barrier dysfunction. Syndets are non-soap surfactants with a pH closer to normal skin, a decreased irritancy potential, a lack of sensitization and the capability to maintain or restore the skin’s acid mantle [47]. The type of surfactant in a cleanser can affect its irritancy potential. Surfactants are needed to lift dirt and oils from the skin, but some types can cause tightness, dryness, irritation and itch after washing [48]. Anionic surfactants are the most common type of surfactants used in cleansers, but they have the greatest irritancy potential, while the nonionic and amphoteric types are less irritating. However, a study conducted in Japan showed that washing with water alone was not inferior to washing with soap for maintaining remission of eczema in pediatric patients with well-controlled AD in Japan [49]. A study by Noviello et al. [50] showed that cleansing with clear water seemed comparable with cleansing with syndet or mild liquid baby cleanser in terms of maintaining the acid mantle and reducing fat content. In light of this evidence, the AAD [12] and JTF [21] guidelines recommend limited use of neutral to low pH, hypoallergenic and fragrance-free soaps or nonsoap cleansers (syndets, aqueous solutions) because of their better tolerance. 

### 3.3. Bath Oils 

Bath oils are valuable for skin care, especially in infants and children. Their use as bathing additives is believed to create a lipid film on the skin surface after the bath [51], acting as a natural skin barrier. A trial conducted in Berlin [52] on children and adults with dry skin investigated the effectiveness of using bath oil additives compared to non-oil-containing skin cleansers for bathing or showering. This study showed that regular bath oil use improves skin barrier function by normalizing the increased TEWL and reducing the loss of natural moisturizing factors. A study demonstrated that using bath oils as early skin care in young infants also decreases the risk of AD development [53]. On the contrary, a review by Shams et al. found no evidence of clinical benefit from including oils as bath additives to treat conditions like atopic eczema [54]. However, it seems the effect of bath oils on skin barrier function is minimal because they do not create a continuous film on the surface. Plenty of different types of bath oils can be used with different capacities to maintain lipid barrier integrity and different capabilities to leave irritating substances on the surface after the bath [55]. Mineral oil is a semi-occlusive ingredient that penetrates the upper layers of the SC. It enhances the skin barrier by reducing the TEWL [56]. Another common product used for the treatment of dermatologic conditions is lanolin. Evidence found that lanolin is useful in recovering the skin barrier and reducing skin bacterial penetration [57]. Furthermore, bathing with mineral oil and lanolin for 10 min daily seems to lead to significant improvement in patients with severe xerosis [58]. Anyway, particular attention must be paid to sensitive skin patients because lanolin can stimulate allergic contact dermatitis [59]. A review conducted on using 17 vegetable oils as therapeutic agents in different skin conditions showed that, in inflammation-affected skin, vegetable oils with unsaturated fatty acids may irritate the skin. Especially, oleic acid (such as olive oil) is an irritant and a well-known penetration enhancer, causing additional structural damage to the SC [60]. In contrast, oils high in linoleic acid (such as sunflower seed oil) and saturated fatty acids (such as coconut oil) might have beneficial effects, including a reduction in *S. aureus* colonization [61]. Natural oils such as evening primrose, sweet almond and jojoba benefit eczema management [62,63,64]. The bath oil containing coconut has shown to be effective in decreasing the severity of the disease and improving barrier function. It also seems to have antimicrobial, antiviral and antifungal activity [65]. A randomized, double-blind controlled trial compared the application of coconut oil and mineral oil, finding that the coconut oil had a significantly better effect on improving skin eczema [66]. Similar results have been seen in studies using sunflower seed and argan bath oils [65]. Soybean oil has been shown to decrease TEWL, increase skin hydration for 20 min every day [29] and reduce topical steroids [67]. Another type of oil studied is oat oil, which has antioxidant, anti-inflammatory and antihistaminic properties [68,69,70]. It also resulted in the recovery of barrier damage in an in vitro model of atopic dermatitis [71] and improved the microbiome composition of the skin barrier [72]. On the contrary, a recent trial randomized 483 children (1–11 years of age) with AD to regular use for 12 months of oily or oat-based emollient bath additives versus standard topical eczema management without bath additives and found no evidence of clinical benefit from including emollient bath additives [30] Many vegetable oils are unstable and degrade by hydrolysis and oxidation, leading to undesirable effects, including microbial growth, especially on infant skin that is undergoing SC maturation, and development of innate immune function [56]. Secondly, an important aspect is the risk of allergic sensitization to food-based skin products, especially when applied through inflamed skin [73,74,75]. A study conducted on 302 children showed a high prevalence of oat sensitization in AD children [76]. In conclusion, further experimental data are required to evaluate the benefit of bath oils in managing AD, considering the different types existing and their risk in terms of potential for allergy. Currently, the crucial step remains the use of emollients after bathing in a bath oil.

### 3.4. Bath Salts 

Bath salts are water-soluble, pulverized minerals added to water during a bath. They are believed to be beneficial in dermatologic disease for removing the dead keratin material and improving the condition of impetiginized or ichthyotic skin. Different studies have highlighted the beneficial effects of dead sea salts [MgCl] in patients suffering from AD [77,78]. The Dead Seas’ water has a very high salt content and is particularly rich in magnesium that binds to water, influencing epidermal proliferation and differentiation and reducing skin inflammation [77,79]. A study conducted on 1408 patients with AD showed complete clearance of skin lesions in 90% of patients after 4–6 weeks of therapy in the Dead Sea area [80]. However, the efficacy of salt baths alone has not been studied systematically in AD. In the current reports, salt baths were investigated with UV therapy. A randomized controlled trial found that synchronous use of narrow-band [NB] UVB treatment and bathing in 10% Dead Sea salt solution was significantly more effective in treating AD than NB UVB monotherapy [80]. Another aspect to consider is that the high concentrations of salt are not easily obtained at home as they require a large amount of salt for every bath. This can lead to corrosion of the bathtub and wastewater pipes, not to mention the impact on the environment. Other types of salts, such as MgSO salt (known as Epsom salt) and NaCl salt, have been studied for their beneficial use [81], but they need further controlled clinical trials to be evaluated as adjunctive therapy in AD. In conclusion, adding salts to water baths may improve skin care in children with AD, but there is still limited scientific evidence recommending their use in bathing.

### 3.5. Rice Starch

Many reports showed that adding rice bran to bath water had beneficial effects on skin erythema, lichenification and itching in patients with AD [82]. A study involving 13 patients with AD aged 19–38 years demonstrated that adding rice starch to bath water (10 g/L, 15 min twice-daily exposure for 4 consecutive days) had a beneficial effect on damaged skin barrier and improved barrier function. The authors hypothesized that the healing effect of starch was because small molecules can penetrate the upper layers of the fissured skin and form a homogenous layer [83]. 

### 3.6. Citric Acid 

Considering the increased pH of the SC in inflamed skin, the acidification of the skin may be a possible preventive or therapeutic strategy in AD. On this line, an intervention study was conducted, dissolving citric acid in tap water until the bath water had a pH of 3.0. The experiment showed that acidic water bathing was effective for severe or refractory AD, and the improvement was demonstrated by obtaining a lower EASI score and TEWL and increased SC hydration [84]. Another beneficial effect of citric acid is that it inhibits the skin’s bacteria proliferation, particularly Pseudomonas. A screening program by Hirotuki et al. found that citric acid suppressed the inflammation induced by applying Pseudomonas ceramidase [85]. However, these studies were conducted on adult patients or animal models. Additional studies on using citric acid on children with AD are required. 

### 3.7. Acetic Acid

There is conflicting evidence on using vinegar as a coadjuvant for treating AD. Studies conducted ex vivo and on murine models have shown that acetic acid has a beneficial effect on reducing eczema [86] and has antimicrobial properties [87]. However, a study examined the effects on Staphylococcus aureus abundance after 14 days of topical dilute apple cider vinegar (0.5% acetic acid) in 11 subjects with AD compared with 11 healthy controls. The results suggested no difference in the mean abundance of *S. aureus* in AD subjects and no significant differences in the skin bacterial microbiome of healthy control subjects [88]. Another recent study showed that diluted apple cider vinegar did not affect skin barrier integrity but caused skin irritation in most subjects [89]. Anyway, the acetic acid effects may depend on the type of vinegar applied and, for its antimicrobial effects, on the type of microorganism involved [90]. Future studies are needed to explore the benefits of vinegar on skin barrier integrity and its use in a safe way.

### 3.8. Other Additives

Other bath additives have been studied to treat AD with less conclusive evidence. In clinical trials, green tea extracts have markedly reduced AD severity and pruritus reduction [91,92]. Similar results were obtained in a study in which 121 patients with recalcitrant AD were instructed to drink oolong tea every day for six months, with a marked to moderate improvement of their condition after one month [93]. These data showed that topical tea during the bath may be an alternative in children with AD. Tannic acid [TA] is a natural agent in grapes and green tea with anti-inflammatory, antioxidant, antimicrobial, antimutagenic and anticarcinogenic activities. In a double-blind cross-over trial, bath additives containing tannic acid improved pruritus in patients with AD without adverse effects [94]. Pine tar and various tar ingredients have also been studied for their antiallergic ingredient. A pilot study by Kam Lun Hon et al. has provided preliminary data on the efficacy of pine tar bath oil as a potential complementary topical treatment [95]. Due to the paucity of data, further evidence-based findings are needed. The therapeutic properties of sodium bicarbonate have been studied to demonstrate its effectiveness against skin pathologies due to its antimicrobial and anti-pruritic properties. A study from a French group suggested that sodium bicarbonate has antimicrobial effects, particularly antifungal activity [96]. 

## 4. Conclusions

Atopic dermatitis is one of the most common cutaneous diseases in children worldwide. Due to its chronic course, the patient and their caregivers need to be adequately educated and confident in the basic management of the disease to gain disease control and improve their quality of life. Effectively managing atopic skin necessitates a multifaceted approach that incorporates a variety of treatment modalities, from simple interventions to more complex measures. The hydration of the skin through emollients represents the cornerstone of the prevention and treatment of atopic dermatitis. At the same time, bathing can have a supporting role if performed correctly. There are still disparate recommendations regarding the frequency, duration and timing of bathing. Daily bathing for about 5–10 min with warm water seems the best option. In addition, there is a crescent interest in the use of bathing additives. They emerged as promising adjunctive therapies with different mechanisms of action, such as anti-inflammatory, antibacterial and anti-pruritus effects, skin barrier repair and restoring the microbiome. The most common additives include bleach baths and baby cleansers, especially in patients with frequent bacterial infections. However, the possibility of the benefit of water baths alone remains unclear. It is possible to conclude that bathing seems to have a favorable risk/benefit balance if performed correctly. Future research can be better designed to provide more conclusive results by standardizing or, at least, documenting the principal bath parameters used in the studies and by using specific and objective measures (for example, SCORAD or EASI) in order to standardize the research’s results. Furthermore, larger sample size and controlled trials are needed to understand better the mechanism of action of these additives, their benefits and the concentration that gives the most clinical benefit without consequences on the health of children with AD.

## Figures and Tables

**Table 2:** Types of additives: why, how and when to use them.

	Why to Use	How to Use	When to Use
Sodium hypochlorite (bleach baths)	Decreasing AD severity thanks to its antimicrobial activity (new studies suggest this effect is obtained at higher concentrations), capacity to modulate the surface microbiome without causing antibiotic resistance, and anti-pruritogenic and anti-inflammatory effects.	Dilute the sodium hypochlorite in bath water (NaOCl 0.005%)	The AAD and European guidelines recommend its use in patients with moderate to severe AD with frequent bacterial infections JTF guidelines [18] are more cautious about its use because it requires further studies
Baby cleansers	Facilitating grease and dirt stripping and helping to maintain or restore the skin’s acid mantle.	Syndets (or non-soap cleansers) with neutral or low pH, hypoallergenic and fragrance-free.	The AAD, European and JTF guidelines recommend their use
Bath oils	Creating a lipid film on the skin after the bath normalizes the increased TEWL and reduces the loss of natural moisturizing factors.	Adding bath oils to the water	The European guidelines recommend their use in the last 2 min of bathing. It is not specified what type of bath oil to use (for example, mineral oil, lanolin, vegetable oil, oat oil)
Bath salt	Removing dead keratin material and improving the condition of impetiginized or ichthyotic skin. Dead Sea Water (MgCl) is the most common type, which contains magnesium that binds to water, influencing epidermal proliferation and differentiation and reducing skin inflammation.	Adding salt to the water	It can be used in combination with NB-UVB treatment Other salts, such as MgSO salt and NaCl salt, need further study to be evaluated as adjunctive therapy in AD
Rice Starch	Improving erythema, lichenification and itching. It is also hypothesized that the starch penetrates fissured skin’s upper layers and forms a homogeneous layer.	10 g/L, 15 min twice-daily exposure for 4 consecutive days	No specific recommendation
Citric Acid	Increasing skin hydration suppresses inflammation and bacterial proliferation (especially Pseudomonas’).	Citric acid dissolved in bathwater	No studies in pediatric age. Further studies are required
Acetic acid (vinegar)	Reducing eczema, probable antimicrobial activity.	Acetic acid dissolved in bathwater	No studies in pediatric age. Further studies are required
Green tea extracts	Reducing AD severity and pruritus.	Topical tea dissolved in bathwater	No studies in pediatric age. Further studies are required
Tannic acid	Anti-inflammatory, antioxidant, antimicrobial, antimutagenic and anticarcinogenic activities.	Tannic acid dissolved in bathwater	No studies in pediatric age. Further studies are required
Sodium bicarbonate	Antimicrobial and antipruritic properties.	Sodium bicarbonate added to bathwater	No studies in pediatric age. Further studies are required

## Data Availability

No new data were created or analyzed in this study. Data sharing is not applicable to this article.
